# Effect of the Teeth Whitening Procedure on the Mineral Composition of Oral Fluid

**DOI:** 10.3390/dj12010009

**Published:** 2023-12-29

**Authors:** Elena A. Ryskina, Oksana A. Magsumova, Mikhail A. Postnikov, Tatiana A. Lobaeva, Bahovaddin B. Ahmedzhanov, Anastasia N. Shishparenok, Dmitry D. Zhdanov

**Affiliations:** 1 Department of Biology and Biotechnology Higher School of Economics, HSE University, 20 Myasnitskaya St., 117418 Moscow, Russia; 2 Department of Therapeutic Dentistry, Samara State Medical University, 89 Chapaevskaya St., 443099 Samara, Russia; oksi63@bk.ru (O.A.M.); m.a.postnikov@samsmu.ru (M.A.P.); 3 Department of Biology and General Genetics, RUDN University, 8 Miklukho-Maklaya St., 117198 Moscow, Russia; lobaeva_ta@pfur.ru; 4 Departments of Fundamental Disciplines Medical University, MGIMO-MED, 3 Novosportivnaya St., 143007 Odintsovo, Moscow region, Russia; 5 Department of Surgery, RUDN University, 8 Miklukho-Maklaya St., 117198 Moscow, Russia; 1032175137@pfur.ru; 6 Laboratory of Medical Biotechnology, Institute of Biomedical Chemistry, 10/8 Pogodinskaya St., 119121 Moscow, Russia; a.shishparyonok@ibmc.msk.ru

**Keywords:** oral fluid, tooth whitening systems, remineralizing therapy, film-forming aerosol, hydrogen peroxide, calcium, phosphorus

## Abstract

The basis of modern tooth whitening systems is the use of a whitening gel, which usually contains hydrogen peroxide or carbamide peroxide. The study included 81 patients aged 22 to 35 years with a tooth color A2 and a darker color on the Vita Classic scale. The purpose of our research was to identify a new approach to whitening teeth to improve safety and gentleness. To perform this, we assessed the effect of the tooth whitening procedure on the mineral composition of the oral fluid. A new approach to the teeth whitening procedure was to use a mouth retractor and a tool for aspirating the whitening gel, which we developed. Before the procedure, a protective film-forming aerosol, which included sodium ascorbate, was applied. After the tooth whitening procedure, the enamel was remineralized with a sealing liquid for 14 days. The concentrations of calcium and phosphorus in the oral fluid were determined using a spectrophotometer with a set of reagents (Human). The results obtained indicate that the new approach to the teeth whitening procedure contributed to less pronounced changes in the concentrations of calcium (+29.07, *p* < 0.001) and phosphorus (−14%, *p* < 0.001) in the oral fluid immediately after the procedure and in combination with the standard procedure for teeth whitening; immediately after this procedure, the calcium concentration increased by 74.4% (*p* < 0.001), and the phosphorus concentration decreased by 23.07% (*p* < 0.001). The use of remineralizing agents led to a faster recovery of the initial levels of calcium and phosphorus in the oral fluid.

## 1. Introduction

Oral homeostasis is contingent upon not only the functionality of oral tissues, anatomical structures within the oral cavity, and the unique characteristics of blood composition but also upon the composition and inherent properties of oral fluid, commonly referred to as mixed saliva. Oral fluid is the least studied of all biological fluids of the human body. Nevertheless, this small secretion volume plays a vital role in keeping oral tissues integrated. A comparative analysis of salivary gland metabolism revealed that its intensity was only marginally lower than that observed in the kidneys and notably greater than that observed in the liver [1,2,3].

Oral fluid comprises a diverse array of well-established and prospective disease biomarkers, leading to its growing utility as a biological fluid for disease diagnosis, for the ongoing assessment of dental health conditions, and for the prediction of disease progression [4,5,6].

Contemporary tooth whitening systems rely on the application of a whitening gel, typically formulated with hydrogen peroxide or carbamide peroxide. Hydrogen peroxide is not a stable compound and decomposes easily. Due to the active forms of oxygen, upon decomposition, hydrogen, hydroxyl, and perhydroxyl radicals are formed. These radicals can affect tooth enamel, causing deproteinization. Whitening gels cleave unsaturated carbon bonds within pigment molecules, thereby reducing their size and complexity, which in turn diminishes absorption and enhances both light reflection and transmission. This process has the potential to remove not only pigment molecules but also mineral elements. This may account for the depletion of calcium and phosphate in these regions and, consequently, the release of minerals into the external environment. This phenomenon is explained by the influence of reactive oxygen species that are generated from bleaching compounds.

Despite the extensive body of scientific research dedicated to the issue of tooth whitening, numerous questions remain unanswered. The currently employed tooth whitening systems and methods for safeguarding whitened teeth lack adequate theoretical substantiation. There are conflicting data on the effect of bleaching factors on the mineral component of oral fluid.

Several studies have demonstrated alterations in mineral metabolism markers due to the influence of various tooth whitening agents [7,8]. Several published sources have proposed that whitening systems do not influence mineral metabolism parameters in mixed saliva and dental hard tissues [9]. The research results obtained are contradictory.

In light of the aforementioned findings, there is a need to investigate the dynamics of biochemical parameters within oral fluid at various observation intervals during professional tooth whitening procedures, with the goal of formulating the most secure approach to mitigating the resultant alterations.

Considering the previously obtained experimental results, a new approach to the teeth whitening procedure was proposed. For this procedure, we used a mouth retractor and a bleaching gel aspiration tool. To ensure a greater degree of safety during the procedure, a protective film-forming aerosol and remineralizing therapy were used.

The mouth retractor we developed will improve the visibility of the working field during the tooth whitening procedure and reduce the risk of peroxide compounds entering the soft tissues of the oral cavity through the use of frames to protect the cheeks and a tongue retainer in the form of a bending bar.

A tool for aspiration of the whitening gel (a hollow tube with a nozzle fabricated of elastic material attached to it) will improve the quality of aspiration, significantly help the collection of oral fluid from hard-to-reach places in the oral cavity, and avoid increased sensitivity of the teeth at the stage of removing the whitening gel.

A protective film-forming aerosol is a composition for spraying onto the gingival surface to protect the mucous membrane of the gum during the tooth whitening procedure. This composition is used as an additional protective agent; it is applied immediately before liquid rubber dams are applied and contains the following components: sodium ascorbate, polyvinylpyrrolidone, distilled water and food flavorings.

Before using the aerosols, the surface of the teeth was covered with polytetrafluoroethylene (PTFE) tape, and then the drug was applied to the surface of the mucous membrane of the gums and into the interdental spaces by directed spraying with several pulses. Aerosols prevent burns to the gum mucosa during the tooth whitening procedure while improving the adhesion of the liquid rubber dam.

It should be noted that most materials used for dental restoration (for example, composite fillings, ceramics, or gold crowns) do not react to whitening agents. Teeth whitening may help increase the release of amalgam components (mercury and silver) after exposure to hydrogen peroxide.

The objective of this research was to study the dynamics of changes in the concentrations of calcium and phosphorus in oral fluid at various time intervals (before and after the tooth whitening procedure) and to determine a safer method with a focus on mitigating the resulting changes in the mineral composition of the oral fluid.

This study suggested that the new approach to tooth whitening will be safer and gentler, which can be confirmed by changes in calcium and phosphorus concentrations over time.

## 2. Materials and Methods

### 2.1. Patients and Whitening Systems

Eighty-one patients participated in the experiment; among them, 43 were women and 38 were men aged 22 to 35 years old with a tooth color A2 and darker according to the Vita Classic scale. The Ethics Committee of Samara Medical University approved this study (protocol 213, 23-04-2020). This protocol complied with the Helsinki Declaration of 1975, as revised in 2000. The protocol was explained to all participants, and after answering every question, patients signed informed voluntary consent to participate in the study and process personal data. Patients with an older oral cavity without general somatic pathology were under observation.

All patients had professional oral hygiene 2 weeks before the office whitening procedure using an ultrasonic device DTE-D7 LED (Woodpecker, Guilin, China) for removal of mineralized dental deposits, an air flow device for removal of pigmented plaque by the air-jet method and polishing with a nylon brush and paste.

The patients were divided into 2 groups (Table 1).

Patients in the first group underwent tooth whitening with the standard Opalescense Boost system (Ultradent Products, Inc., South Jordan, UT, USA) using a gel based on 40% hydrogen peroxide. Patients in the second group underwent tooth whitening with the Opalescense Boost system (Ultradent Products, Inc., South Jordan, UT, USA) with modifications.

The tooth whitening system included 2 syringes with a whitening gel and an activator, which were mixed together before the procedure. A whitening gel is a chemical activation gel containing 40% hydrogen peroxide. It also contained 5% potassium nitrate and 0.22% sodium fluoride, which are necessary to reduce tooth sensitivity during and after the whitening procedure. The kit included an Opal Dam Green liquid dam to protect the soft tissue and gingival margin, as well as a bite block (Iso Block) for support and fixation of the tongue.

Stages of tooth whitening:

1. Patients had oil with vitamin E applied to their lips, a YDM cheek retractor (YDM, Tokyo, Japan) was placed, and the working field was isolated with cotton swabs, which were placed in the area of the upper and lower vestibule of the oral cavity.
2. A liquid rubber dam was applied in a layer of at least 2 mm to the marginal part of the gum mucosa and 0.1–0.2 mm of the cervical area of the teeth, after which photopolymerization was carried out.
3. Whitening gel was applied to the vestibular surface of 20 teeth up to the 2nd premolar of each quadrant for 20 min. After each session, the gel was removed using a vacuum cleaner and cotton rolls.
4. The procedure for applying the gel was repeated three times.
5. After completing the last cycle, the whitening gel was washed off with water, and the insulation and the YDM cheek retractor were removed.

Stages of tooth whitening with modifications:

To prevent discomfort and pain, all patients in the second group additionally received Relief ACP gel (Philips, Cambridge, MT, USA) in an individual mouthguard for 10 min immediately before the procedure. This gel contains 5% potassium nitrate, which penetrates the dentinal tubules and has membrane-depolarizing potential, and 0.22% sodium fluoride, which obstructs the dentinal tubules. Following the procedure, remineralization therapy was performed using enamel-sealing liquid (HumanChemie, Alfeld (Leine), Germany) over the course of 14 days.

1. Patients had oil with vitamin E applied to their lips, and a retractor developed by us was installed to protect the soft tissues of the oral cavity; the working field was isolated with cotton rolls, which were placed in the area of the upper and lower vestibule of the oral cavity.
2. We applied a film-forming aerosol to the marginal part of the gum mucosa and 0.1–0.2 mm of the cervical area of the teeth, followed by the application of a liquid rubber dam in a layer of at least 2 mm, after which photopolymerization was carried out.
3. Whitening gel was applied to the vestibular surface of 20 teeth up to the 2nd premolar of each quadrant for 20 min. After each session, the gel was removed using our proprietary whitening gel aspiration tool and cotton swabs.
4. Three sessions of 20 min each were conducted.
5. After completing the last cycle, the whitening gel was washed off with water, the insulation and mouth seal were removed, and an enamel-sealing liquid was applied to the teeth.
6. Every day for 14 days, once a day, 15-min applications of the enamel-sealing liquid were carried out.

### 2.2. Preparation of Oral Fluid Samples and Determination of Calcium and Phosphorus Concentrations

Patients provided oral fluid in a test tube, and the tube with the resulting sample was frozen at −20 °C. To determine the indicators in the oral fluid, the test tube containing the sample was thawed at +3 °C for 10 h, after which the sample temperature was increased to room temperature. The determination of the mineral composition of the oral fluid was carried out at the Institute of Biomedical Chemistry. Before determining the content of mineral elements, tubes with oral fluid samples were centrifuged for 5 min on an Eppendorf MiniSpin centrifuge at a speed of 5000× *g*.

To assess the effect of the tooth whitening procedure, the mineral composition of the oral fluid was determined before the procedure and immediately after, 14 days after and 30 days after the procedure on an Aquarius CE 7200 spectrophotometer (Cecil Instruments Ltd., Peterborough, UK). The concentrations of calcium were measured photometrically with o-cresolphthalein complexone [10], and the phosphorus content was measured photometrically with an antilipid factor [11]. To determine the concentration of mineral elements, human kits (HumanChemie, Alfeld (Leine), Germany) and Humatrol control serum (Human, Germany) were used.

### 2.3. Data Analysis and Processing

The analytical software SPSS 25 (IBM SPSS Statistics, Armonk, NY, USA) was used for the data analysis. M ± SD was used to describe the results, where M is the arithmetic mean, SD is the standard deviation, and Δ% is the difference in percentage from the average sample values (Mtest/Mcontrol) × 100%).

Mtest—indicator value before the procedure
Mcontrol—indicator value after the procedure
+ increase in indicator relative to the initial value before the procedure
− decrease in the indicator in relation to the initial value of the indicator before the procedure

Comparisons between independent groups were performed using Kruskal-Wallis analysis followed by pairwise comparisons using the Mann-Whitney test. Differences were considered to be statistically significant at *p* < 0.05.

## 3. Results

The calcium content in saliva ranges from 0.75 to 3.0 mM (the same as that in plasma). Calcium can be ionized (Ca^2+^) or protein-bound. After the in-office tooth whitening procedure, both group 1 and group 2 patients had increased calcium ion concentrations in their oral fluid (Table 2).

When comparing the calcium concentration in the oral fluid immediately after the tooth whitening procedure, a statistically significant difference in the level of the macronutrient in the oral fluid was observed between the two groups (Table 3).

The results obtained showed a decrease in the phosphorus content in the oral fluid after the tooth whitening procedure in patients in both groups (Table 4).

There was no significant difference in the phosphorus concentration (*p* = 0.238) after the tooth whitening procedure between the first and second groups, in contrast to the change in the calcium concentration.

## 4. Discussion

Bone tissue is involved in the systemic regulation of calcium metabolism. There are two fractions of calcium in bone tissue: labile and stable. The labile fraction of bone actively exchanges calcium with oral fluid. There is also bidirectional calcium exchange between labile and stable fractions, which is controlled by factors responsible for bone tissue remodeling.

Under natural conditions, the source of calcium, phosphorus, and fluoride for tooth enamel is oral fluid, which is oversaturated with almost all forms of calcium phosphate. Calcium phosphates play an active role in the mineralization of hard dental tissues. Thus, the chemical composition of hard dental tissues depends on the composition of the surrounding oral fluid.

The data obtained indicate a significant increase in the concentration of calcium ions in the oral fluid after the tooth whitening procedure in the first group (74.4%) compared with the initial value. The new approach to the tooth whitening procedure resulted in a less significant increase in calcium levels after the tooth whitening procedure (29.1%). The results indicate that calcium ions are released from the enamel crystal lattice, which leads to an increase in their concentration in the oral fluid. Calcium concentrations returned to baseline levels after 30 days in both the first and second groups of patients.

In the work of Uspenskaya O.A. et al. [12], an increase in the level of calcium in the oral fluid was observed during professional tooth whitening, which indicates the release of calcium from the enamel layers. An increase in the level of calcium and a decrease in the concentration of phosphorus in the oral fluid were established after the tooth whitening procedure with the Opalescense Boost, ZOOM Advance POWER, and ZOOM Phillips White Speed systems [13].

Several studies have indicated that tooth whitening leads to a decrease in the calcium and fluoride contents of the hard tissues of teeth [14,15]. Several studies have reported that no changes in dental hard tissues occur after professional or at-home tooth whitening procedures [16,17]. In the studies of F.Ya. Shakira et al. reported that tooth whitening systems reduce the levels of calcium and potassium in tooth enamel, and the levels of F and O in the enamel increase [18].

Immediately after the tooth whitening procedure, a decrease in the concentration of phosphorus in the oral fluid was observed in both the first group (23.07%) and the second group (14%). In the first group of patients, more significant changes in phosphorus concentration were detected; these changes persisted for 14 days, after which the phosphorus concentration returned to the original level after 30 days. In the second group, no changes in phosphorus concentration were observed after 14 days. The use of a combination of prophylactic agents in the second group led to a smaller decrease in the level of phosphorus in the oral fluid and a rapid restoration of the initial level of this microelement.

Several publications have reported an increase in the phosphate content in oral fluid, while other studies have shown that the content of this trace element remains unchanged.

A study by Carmen Llena et al. assessed calcium and phosphorus levels in enamel and dentin after teeth whitening with 37.5% hydrogen peroxide and 35% carbamide peroxide. It was shown that the content of calcium and phosphorus decreased in the dental hard tissues of teeth, but these two parameters did not significantly differ between the treated and control samples [19].

V. Cavalli et al. showed that high concentrations of hydrogen peroxide can lead to a decrease in the mineral content of Ca and P and a decrease in the microhardness of tooth enamel [20,21]. Coceska E. et al. confirmed that highly concentrated bleaching agents can damage the enamel surface. On the other hand, they also showed that enamel changes are reversible and can be reversed with remineralization therapy [22].

In studies by Klaric E. et al., an increase in the concentration of phosphate c in the control group was detected using FT-Raman spectroscopy. The control group samples were stored in a remineralizing solution to simulate clinical conditions. A study showed that storing samples in a remineralizing solution promoted the deposition of calcium and phosphorus ions on the enamel surface [23].

Pinto Abd. et al. evaluated whitening systems with high concentrations of hydrogen peroxide, including the Opalescent Boost system. Enamel microbiopsy was assessed using total reflection X-ray fluorescence (TXRF) and colorimetric spectrophotometry (SPEC) before, after and 14 days after the whitening procedure [24]. Some authors believe that the effects of hydrogen peroxide breakdown products on tooth enamel during tooth whitening can lead to the loss of mineral components [25,26].

In particular, in the work of M. Fittler et al. [27], it was demonstrated that tooth whitening products containing hydrogen peroxide may cause undesirable side effects, including damage to the morphology and structure of the tooth. One of the most common side effects of peroxide-based bleaching, especially when peroxide is used at high concentrations, is hyperesthesia, which can occur due to the release of oxidative free radicals. Additionally, it was shown that in-office tooth whitening using peroxides can cause side effects such as hyperesthesia and damage to the natural organic matrix of enamel and dentin [28].

In the work by Benahme A. et al. [29], several critical issues were observed for tooth whitening systems containing high concentrations of hydrogen peroxide and carbamide peroxide. These systems can lead to deproteinization and demineralization of teeth, which is associated with oxidation processes and can cause a number of side effects.

New, simple, and safe tooth whitening recommendations have become key. For example, a new technique using modified titanium dioxide nanoparticles activated by blue light allows you to safely whiten teeth without damaging the enamel [30]. Compared with classic whitening agents (peroxide, hydrogen peroxide, and carbamide peroxide), titanium dioxide nanoparticle whitening not only has a similar whitening effect but also causes significantly less damage to the enamel structure.

Ferreira A.F.M. et al. [31] tested the hypothesis that the contents of enamel components (mineral, organic and water content, as well as the permeability of tooth enamel) change after a short whitening procedure from the outer layer to the inner layer of tooth enamel. Changes in enamel composition were significant only in the outermost layers of enamel. It has been shown that as the level of minerals decreases, the content of organic components and water increases, and the permeability of tooth enamel decreases.

The tooth samples were divided into three groups and subjected to a whitening procedure using the following systems: LP15% (15% Lase Peroxy Lite), LP25% (25% Lase Peroxy Sensy) and LP35% (35% Lase Peroxy Sensy), with different concentrations of hydrogen peroxide. Bleached tooth samples were also irradiated with a laser light source. After bleaching, all the samples were evaluated using scanning electron microscopy. All the whitening gels caused the same color change (*p* > 0.05). The LP25% and LP35% whitening systems significantly reduced the hardness of the tooth enamel after whitening. It was noted that there was a progressive trend toward a greater percentage decrease in tooth enamel hardness with increasing hydrogen peroxide concentration [32].

In our study, a statistically significant difference was found in the calcium concentration in the oral fluid after the whitening procedure between the two tooth whitening systems tested (*p* = 0.012). There was no significant difference in the concentration of phosphate in the oral fluid during tooth whitening between the first and second groups of patients (*p* = 0.238).

## 5. Conclusions

Whitening agents affect mineral metabolism, particularly the levels of calcium and phosphorus, which should be taken into account when carrying out tooth whitening procedures.

However, the use of an oral retractor, an instrument for aspirating bleaching gel, a protective film-forming aerosol, and 14 days of remineralization therapy with enamel sealing fluid resulted in less significant changes in calcium and phosphorus concentrations and contributed to a more rapid restoration of the initial levels of these mineral elements in the oral fluid.

The mouth retractor we developed to protect the oral cavity allowed us to improve the visibility of the working field during the in-office tooth whitening procedure and reduce the risk of peroxide compounds entering the soft tissues of the oral cavity. Improving the safety and quality of the tooth whitening procedure was also achieved by improving the ergonomics and manufacturability of the instrument for aspirating the whitening gel. An additional protective agent (aerosol), applied immediately before using the liquid rubber dam, contains the physiological antioxidant sodium ascorbate and polyvinylpyrrolidone, which have a high complexing ability, allowing increased elongation of the antioxidant action. The use of a film-forming aerosol helped protect the gum mucosa from peroxide compounds during the tooth whitening procedure while improving the adhesion of the liquid rubber dam.

Redineralizing therapy with enamel-sealing liquid is known to provide deep fluoridation, reliable protection and restoration of tooth enamel.

After this therapy was administered, the phosphorus content in the oral fluid was restored more quickly after the tooth whitening procedure, unlike the calcium content.

Our study confirmed that adhesive restorations should not be performed on patients less than a month after tooth whitening.

The materials from our study confirmed that adhesive restorations should not be performed on patients earlier than one month after tooth whitening.

During the tooth whitening procedure, a set of preventive measures should be used to prevent fluctuations in mineral elements when using whitening systems. A new approach to whitening teeth, using the modifications we proposed, was found to be safer and gentler. Thus, the hypothesis we proposed was confirmed.

## Figures and Tables

**Table 1:** Distribution of patients by group, age, and sex.

Group/Number of Patients	Gender	Number of Patients
First group *n* = 41	W	23
	M	18
Second group *n* = 40	W	21
	M	19

**Table 2:** Effect of the tooth whitening procedure on the concentration of calcium in the oral fluid (mM).

Calcium Concentration	Before the Procedure	After the Procedure	After 14 Days	After 30 Days
Office teeth whitening (group 1)				
M ± SD	2.19 ± 0.16	3.82 ± 0.24	2.95 ± 0.18	2.14 ± 0.13
Δ%	-	+74.4%	+34.7%	−2.3%
*p*	-	<0.001	<0.01	0.553
Office teeth whitening with modifications (group 2)				
M ± SD	2.30 ± 0.09	2.97 ± 0.11	2.44 ± 0.11	2.20 ± 0.09
Δ%	-	+29.1%	+11.4%	−4.3%
*p*	-	<0.001	<0.01	0.237

M—mean value; SD—standard deviation; Δ%—difference in percentage of sample mean values.

**Table 3:** Comparison of calcium concentrations in the oral fluid immediately after the tooth whitening procedure between the first and second groups of patients (mM).

Calcium Concentration	Office Teeth Whitening (Group 1)		Office Teeth Whitening with Modifications (Group 2)		Comparison of Two Systems (*p*-Level)
	Before	After	Before	After	
M ± SD	2.19 ± 0.16	3.82 ± 0.24	2.30 ± 0.09	2.97 ± 0.11	
Δ%	-	+74.4%	-	+29.1%	
*p*	-	<0.001	-	<0.001	0.012

M—mean value; SD—standard deviation; Δ%—difference in percentage of sample mean values.

**Table 4:** Effect of the tooth whitening procedure on the concentration of phosphorus in oral fluid (mM).

Phosphorus Concentration	Before the Procedure	After the Procedure	After 14 Days	After 30 Days
Office teeth whitening (group 1)				
M ± SD	4.45 ± 0.22	3.43 ± 0.18	3.88 ± 0.17	4.44 ± 0.22
Δ%	-	−23.07%	−13%	−0.3%
*p*	-	<0.001	<0.01	0.674
Office teeth whitening (group 2)				
M ± SD	4.45 ± 0.21	3.83 ± 0.21	4.46 ± 0.21	4.41 ± 0.20
Δ%	-	−14%	+0.22%	−0.9%
*p*	-	<0.001	*p* > 0.05	0.221

M—mean value; SD—standard deviation; Δ%—difference in percentage of sample mean values.

## Data Availability

The data presented in this study are available upon request from the corresponding author. The data are not publicly available due to ethical restrictions.
